# Robotic operations in urgent general surgery: a systematic review

**DOI:** 10.1007/s11701-022-01425-6

**Published:** 2022-06-21

**Authors:** Alexander Reinisch, Juliane Liese, Winfried Padberg, Frank Ulrich

**Affiliations:** 1 Department of General, Visceral and Oncologic Surgery, Wetzlar Hospital and Clinics, Forsthausstr. 1, 35578 Wetzlar, Germany; 2 Department of General, Visceral, Thoracic, Transplant and Pediatric Surgery, University Hospital Giessen, Giessen, Germany

**Keywords:** General surgery, Minimally invasive surgery, Robotic surgery, Emergency surgery, Urgent surgery

## Abstract

Robotically assisted operations are the state of the art in laparoscopic general surgery. They are established predominantly for elective operations. Since laparoscopy is widely used in urgent general surgery, the significance of robotic assistance in urgent operations is of interest. Currently, there are few data on robotic-assisted operations in urgent surgery. The aim of this study was to collect and classify the existing studies. A two-stage, PRISMA-compliant literature search of PubMed and the Cochrane Library was conducted. We analyzed all articles on robotic surgery associated with urgent general surgery resp. acute surgical diseases of the abdomen. Gynecological and urological diseases so as vascular surgery, except mesenterial ischemia, were excluded. Studies and case reports/series published between 1980 and 2021 were eligible for inclusion. In addition to a descriptive synopsis, various outcome parameters were systematically recorded. Fifty-two studies of operations for acute appendicitis and cholecystitis, hernias and acute conditions of the gastrointestinal tract were included. The level of evidence is low. Surgical robots in the narrow sense and robotic camera mounts were used. All narrow-sense robots are nonautonomous systems; in 82%, the Da Vinci^®^ system was used. The most frequently published emergency operations were urgent cholecystectomies (30 studies, 703 patients) followed by incarcerated hernias (9 studies, 199 patients). Feasibility of robotic operations was demonstrated for all indications. Neither robotic-specific problems nor extensive complication rates were reported. Various urgent operations in general surgery can be performed robotically without increased risk. The available data do not allow a final evidence-based assessment.

## Introduction

Laparoscopic interventions are established in general surgery for urgent, acute diseases of the abdominal organs. For acute appendicitis and cholecystitis, laparoscopic operations are the standard of care and are recommended in guidelines [1, 2]. Many surgical departments also operate on gastrointestinal perforations, incarcerated hernias and bowel obstruction laparoscopically on a regular basis.

However, robotic surgery has been an integral part of general surgery for over two decades and is becoming increasingly widespread. From 2012 to 2018, an increase in general surgical robotic interventions by more than a factor of 8 was described, reaching up to 15.1% of all general surgical operations in the USA [3]. This raises the question of the significance of robotic surgery in urgent surgery. Recently, this led to the publication of a position paper by the World Society of Emergency Surgery (WSES) [4].

We can distinguish two groups of robots as follows: surgical robots (SRs) in a narrower sense and robotic camera mounts (RCMs). SRs in the stricter definition are nonautonomous systems, which are controlled by a surgeon via a console. The main representative of this group of robots is the Da Vinci^®^ system from Intuitive Surgery Inc. (Sunnyvale, CA, USA) since the products from Computer Motion (ZEUS, AESOP; Santa Barbara, CA, USA) were discontinued after Computer Motion and Intuitive merged in 2003. Recently, the Da Vinci^®^ system has faced competition from other manufacturers (e.g., CMR Surgical Versius^®^, Cambridge, UK; Hugo^®^, Medtronic, Dublin, Ireland; and Microhand S, China; Dexter^®^, Distalmotion, Switzerland).

A number of advantages are accredited to robotic surgical systems as follows: systems such as the Da Vinci^®^ system should help to overcome the disadvantages of laparoscopic surgery, such as physiological tremors and restricted degrees of freedom. RCMs, such as Soloassist^®^ II (AKTORmed, Barbing, Germany), promise the liberation of the physician acting as a surgical assistant. However, these operations are very similar to “classic” laparoscopy. All robotic systems also advertise improved ergonomics and optimized visualization. Whether these properties lead to advantages over traditional laparoscopy is the subject of debate. All robotic systems are theoretically predisposed for technical malfunctions that may cause harm to the patient. Several publications report on better outcomes of robotic surgery, e.g., conversion rates, morbidity (including postoperative ileus) and postoperative stay in colorectal surgery [5, 6]. However, almost all publications examine elective operations. Against the background and known advantages of laparoscopic operations in urgent operations, the possible benefit of surgical robots in urgent general surgery must be examined and discussed. The aim of this review is to analyze and classify the available data on this topic.

## Methods

### Literature search

Two of the authors (AR and JL) independently searched PubMed (1980-present) and the Cochrane Library (1980-present). The systematic review was performed according to the preferred reporting items for systematic reviews and meta-analysis (PRISMA) guidelines [7].

A two-stage analysis was conducted. First, a preliminary search and screening of 500 results was performed on October 26th and 27th, 2020, to elaborate the principles of the further analysis. The results of this first search indicated that the number of publications of interest was very limited. Therefore, we decided to include case reports and case series in the systematic analysis. Furthermore, this first search showed a massive heterogeneity of data. To give the reader a full overview of the topic, we decided not to attempt a meta-analysis since a meta-analysis would have limited the useable studies to only a few. A strict systematic review or a meta-analysis would necessarily have meant that most urgent surgical diseases could not have been analyzed. The primary goal of this work, however, was to cover robotic-assisted operations in urgent surgery as broadly as possible, which was only possible through a partially descriptive evaluation.

The second definitive search was run on December 31st, 2021.

The following search terms were used in combination with “robotic” and “robotic surgery”: adhesiolysis, appendectomy, appendicitis, bowel obstruction, cholecystectomy, cholecystitis, diverticular disease, diverticulitis, hernia, incarceration, perforation, peritonitis, ulcer, urgency and emergency (Appendix 1).

The search terms were partially truncated to include as many grammatical variables as possible.

### Inclusion criteria

All studies, case reports and case series describing urgent general and visceral surgical procedures in connection with robotic surgery were included. Manuscripts were included in which typical general or visceral surgical operations were described; inclusion was not decided by which specialty the operation performed. If study populations were published multiple times, the more recent publication was included.

### Exclusion criteria

Publications that did not report the original data were excluded, as were those with overlapping study populations (see above). Urological, gynecological or vascular surgery emergency interventions were excluded. Reports on the thoracoscopic robotic treatment of diaphragmatic hernias were also excluded. If the full text was not available, the study was also excluded.

### Evaluation

If the specified information was extractable, complications were classified according to the Clavien–Dindo classification, whenever applicable [8]. In all other cases and biliary complications in cholecystectomies, the complications were mentioned separately.

### Outcomes of interest

All included manuscripts were examined with a focus on the following factors: (a) primary objective of the study and technical aspects, (b) complications (see above) and whether these complications could be related to the use of the robot, (c) pros and cons of the use of the robotic operation, (d) financial aspects, (e) factors related to the acute/urgent situation and (f) further outcomes of interest.

## Results

**Fig. 1:**
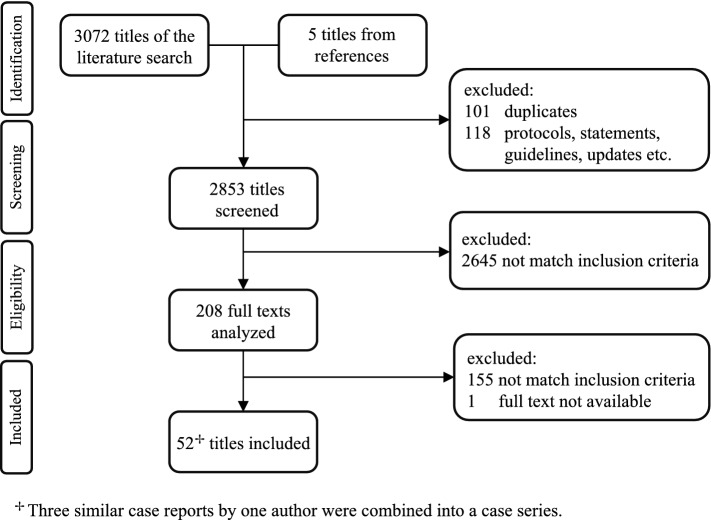
PRISMA-Flow diagram showing the literature search and the study selection with 52 relevant studies enrolled in this review

The literature search resulted in a total of 3072 (+ 5, see below) findings, of which 219 were eliminated (duplicates, letters, replies, guidelines, etc.). A total of 2853 papers were analyzed. The review of the references of the analyzed full texts led to the inclusion of five additional studies. A total of 2645 publications were excluded since the title and/or abstract did not meet the inclusion criteria. The full texts of 208 studies met the criteria and were further analyzed. No unpublished studies were obtained. The full-text analysis revealed that 155 of these studies did not contain data of interest for this review, and one full text was unavailable. Three similar case reports were combined into a case series [9–11]. In total, 52 studies were included, five of which were evaluated for more than one indication (Fig. 1) [12–16]. Apart from one paper on a spleen hematoma, all reports on urgent robotic operations could be assigned to four classic general surgical fields: appendectomies, cholecystectomies, hernias (partly with intestinal obstruction) and gastrointestinal procedures [15].

Of the included papers, 15 were case reports or case series. None of the studies were randomized, and 24 were controlled. Six studies were prospective, and the remaining studies were retrospective [17–22]. Only four retrospective and none of the prospective studies explicitly examined urgent robotic operations, and two of these were controlled studies [23–26].

Two studies focused on the use robotic camera mounts (RCM), and they included 302 urgently operated patients [14, 21]. All other 50 studies examined the use of surgical robots in the narrow sense (SR). In these studies, 955 urgently operated patients were included. A total of 655 of these patients (68.6%) were treated using the Da Vinci^®^ system (41 studies, 82% of the SR studies). Hosein et al. and Gangemi et al. did not specify the type of SR, and the 261 patients included in their studies accounted for 27.4% of the SR patients [27, 28]. An alternative SR was utilized in only 39 operations (3.6%).

Complication rates were listed in all but one study, but with varying degrees of accuracy [29]. Some studies reported complications only above a certain degree of severity, i.e., Clavien–Dindo grade ≥ 3. It should be noted that only 22 of the 52 studies differentiated whether the complication occurred in the subgroup of emergency robotic procedures, and 12 of these were case reports. In most of the included studies, only the complication rate of the entire robotic group was listed. In addition, there was no strict distinction between intra- and postoperative complications, while the follow-up periods differed greatly.

Since urgent operations were neither an interest, endpoint nor variable in the majority of the studies, evidence grading, e.g., the Newcastle–Ottawa Quality Assessment Scale, cannot be applied meaningfully. Regarding urgent operations, the levels of evidence for almost all included studies must be assessed as low, which was true particularly for case series and case reports. One paper was excluded because it was a duplicate publication of a more or less identical patient group [26].

### Appendectomies

Seven papers reported on urgent appendectomies using robotic surgery in a total of 196 patients (Table 1). The vast majority of patients (*n* = 185) were analyzed in two studies, both using RCM. Three more publications were case reports or case series. As a result, only 11 patients were urgently appendectomized using an SR. No complications or conversions were reported.

**Table 1:** Appendicitis

References	Study design						Outcome			Further notes
	Design	Study period	*n* (urgent, robotic)	*n* (non-urgent)	Primary study objective	Robot	Differentiated urgent vs. non-urgent	Complications conversions	Further outcomes of interest	
				*n* (other)						
Cadiére et al. [16]	R-NRNC	3/1997–2/2001	1	–	Feasibility	Da Vinci	No	No complications		See also *cholecystitis*
				145				No conversions		
Kelkar et al. [22]	P-NRNC	3/2019–4/2019	4	–	Feasibility	Versius	No	No complications		
				26				No conversions		
Kibar et al. [51]	CR	n.d	1	–	–	Da Vinci	Yes	No complications		Appendicovesical fistula
				–				No conversions		
Mittal et al. [21]	P-NRNC	n.d	22	–	Feasibility/usefulness	FreeHand RCM	No	No complications	Liberation of the surgical assistant	
				–				No conversions		
Ohmura et al. [14]	R-NRC	12/2014–3/2017	163	3	feasibility	Soloassist RCM	No	“No device-related complications”	Liberation of the surgical assistant	See also *cholecystitis* and *hollow organs*
				783				No conversions		
Yi et al. [12]	CS	3/2014	2	–	Feasibility	Micro Hand S	Yes	No complications		See also hollow organs
				1				No conversions		
Yi et al. [13]	CS	4/2014–5/2014	3	1	Feasibility	Micro Hand S	No	No complications	2 patients withdrawn	See also cholecystitis and *hollow organs*
				6				No conversions		

*CR* Case report, *CS* Case series, *n.d.* not documented, *P-NRNC* prospective, non-randomized, non-controlled, *R-NRC* retrospective, non-randomized, controlled, *R-NRNC* retrospective, non-randomized, non-controlled, *RCM* Robotic camera mount

The three case reports/case series specified closure of the appendix stump (sutures), and the other studies did not provide any information on this. There were no reports of robotic single-incision appendectomies.

### Cholecystectomies

Thirty publications reported on urgent robotic cholecystectomies, none of which were randomized (Table 2). Three studies were prospectively controlled, and 14 were retrospectively controlled. Of the uncontrolled studies, three were prospective, and six were retrospective. Four case reports or case series have been summarized in the table for better clarity [13, 30–32].

**Table 2:** Cholecystitis

References	Study design						Outcome			Further notes
	Design	Study period	*n* (urgent, robotic)	*n* (non-urgent)	Primary study objective	Robot	Differentiated urgent vs. non-urgent	Complications conversions	Further outcomes of interest	
				*n* (other)						
Ayloo et al. [52]	R-NRC	9/2005–6/2012	45	134	MIRC vs. MILC	Da Vinci	No	1.7% CD ≥ 3; 1.7% CD < 3”	3 conversions in MILC; OT longer in MIRC	
				147				no conversions		
Balachandran et al. [53]	R-NRC	10/2011–7/2014	76	339	SIRC vs. MILC	Da Vinci	No	1% ileus 0.2% bile leakage	6.5% hernias in SIRC LOS shorter SIRC	All performed by 1 surgeon
				263				2.9% to MILC, 3.2% to open		
Bibi et al. [33]	R-NRNC	6/2012–1/2013	31	71	Safety of SIRC	Da Vinci	No	4% CD < 3	50% of conversions due to inflammation	
				–				3.9% conversions		
Buzad et al. [17]	P-NRC	1/2012–5/2012	2	18	SIRC vs. SILC	Da Vinci	No	No complications	No differences in OT and costs	SILC reviewed retrospectively
				10				No conversion		
Cadiére et al. [16]	R-NRNC	3/1997–2/2001	4	44	Feasibility	Da Vinci	No	25% (*n* = 1) CD 2		See also *appendicitis*
				98				no conversions		
Chung et al. [29]	R -NRC	8/2013–1/2015	7	63	SILC vs. MILC	Da Vinci	No	n.d	2.8% 30 d readmission	
				70				1.4% conversion to open		
Daskalaki et al. [54]	R-NRNC	7/2011–2/2013	28	156	ICG Cholangio-graphy	Da Vinci	No	1% CD ≥ 3; 2.2% CD < 3	cholangiography > 94%	
				–				no conversions		
Gangemi et al. [28]	R- NRC	2008–2015	130	546	Risk factors for conversion	n.d	N.d	n.d		
				289				0.15% (*n* = 1) conversions		
Giulianotti et al. [15]	R-NRNC	10/2000–11/2002	7	45	Descriptive; RC vs. LC	Da Vinci	No	1.9% (*n* = 1) complications n.d		
				123				1.9% (*n* = 1) conversions		
Gonzalez et al. [55]	R-NRC	1/2012–9/2012	20	146	SILC vs. SIRC vs. SPIDER	Da Vinci	No	1.2% CD ≥ 3; 0.6% CD < 3	OT for SIRC longer than SILC/SPIDER	
				335				No (3 additional incisions)		
Honaker et al. [56]	R-NRC	3/2013–2/2014	1	17	MIRC vs. MILC	Da Vinci	No	No complications	LOS shorter MIRC	
				40				No conversions		
Jeong Jang et al. [57]	R-NRNC	4/2019–8/2020	2 (5)	72 (69)	Feasibility of SIRC	Da Vinci	No	1.35% CD 1		3 acute cholecystitis diagnosed intraoperatively
				–				No conversions		
Kalteis et al. [58]	R-NRC	4/2002–11/2004	20	52	Safety and effectiveness	AESOP	No	1.4% CD < 3	Liberation of the surgical assistant	
				72				No conversions		
Konstantinidis et al. [18]	P-NRNC	3/2011–7/2011	4	41	Feasibility and effectiveness	Da Vinci	No	2.2.% CD 3b, 5.3% < CD 3	20% intraoperative gallbladder-ruptures	
				–				No conversions		
Kornprat et al. [19]	P-NRC	2001–2006	2	18	MIRC vs. MILC	Zeus	No	No complications	OT longer in MIRC	
				26				No conversions		
Kubat et al. [23]	R-NRC	5/2012–8/2013	74	76	Urgent vs. non-urgent MIRC	Da Vinci	Yes	12% complications in urgent, incl. 1.5% (*n* = 1) BD injury	LOS longer in urgent, overall complications + SSI no differences	
				–				1.5% (*n* = 1) conversions		
Kulaylat et al. [36]	R-NRC	1/2015–12/2018	10	69	SIRC/MIRC vs. SILC/MILC	Da Vinci	No	“No differences in complications”	OT longer in robotic, costs higher in robotic	Pediatric patients
				220				No conversions		
Li et al. [42]	R-NRC	8/2013–12/2015	17	61	Safety, resources SIRC vs. MILC	Da Vinci	No	3.8% < CD 3 (20% in MILC)	1.9% conversion rate in MILC	OT, LOS and costs higher in SIRC
				367				No conversions		
Mattei et al. [59]	R-NRNC	2013–?	1	19	Feasibility in pediatric patients	Da Vinci	No	20% seromas (CD < 3)	OT longer, LOS shorter	
				–				No conversions		
Ohmura et al. [14]	R-NRC	12/2014–3/2017	101	172	Feasibility	Soloassist RCM	No	“No device-related complications”	LOS shorter; liberation of surgical assistant	See also *appendicitis* and *hollow organs*
				848				No conversions		
Rosales-Velderrain et al. [60]	P-NRNC	3/2013–5/2015	4	10	Safety, feasibility in pediatric patients	Da Vinci	No	7% (*n* = 1) seroma		
				–				No conversions		
Schertz et al. [35]	R-NRC	8/2013–4/2018	3/6	101/99	SIRC vs. MIRC	Da Vinci	No	1 enterotomy (MIRC), 2.9% hernias (SIRC)	OT, LOS in SIRC shorter	
				–				Excluded		
Su et al. [61]	R-NRC	2/2014–9/2015	10	41	SIRC vs. SILC	Da Vinci	No	No complications	OT in urgent longer Pain lower in SIRC	
				63				No conversions		
Svoboda et al. [20]	P-NRC	11/2012–2/2014	159	112	SIRC in BMI ≥ 30 vs. < 30	Da Vinci	No	0.9% (*n* = 1), incisional hernia	OT longer in obese patients	Inconclusive data
				–				no conversions		
Tao et al. [62]	R- NRC	1/2006–2/2020	13	161	MILC vs. MIRC	Da Vinci	No	12.3% overall in RC	RC vs. LC CD 1 > , CD 2 < , CD 3 equal, no CD IV	All acute cholecystitis diagnosed intraoperatively
				441				No conversion		
Vidovszky et al. [34]	P-NRNC	1/2012–1/2013	13	82	apPlicability of SIRC	Da Vinci	No	4.2% CD 3; 2.1% < CD 3	One disruption of DC 4 technical problems	5 inflammations diagnosed intraoperatively
				–				8.4%; 1 due to inflammation		
Case Series Case Reports	CS/CR	2001–2021	11		Feasibility	Da Vinci (3), Microhand S (1)	No	No complications	Bustos et al. [31]; Hanisch et al. [30]; Milone et al. [32]; Yi et al. [13]	
								No conversions		

Only statistically significant differences listed

*BMI* Body mass index, *CD* Clavien-Dindo classification of complications [8], *CR* Case report, *CS* Case series, *DC* Ductus cysticus, *LC* laparoscopic cholecystectomy, *LOS* length of stay, *MILC* Multi-incision laparoscopic cholecystectomy, *MILC* Multi-incision robotic cholecystectomy, *n.d.* not documented, *OT* Operation Time, *P-NRC* prospective, non-randomized, controlled, *P-NRNC* prospective, non-randomized, non-controlled, *RC* Robotic cholecystectomy, *R-NRC* retrospective, non-randomized, controlled, *R-NRNC* retrospective, non-randomized, non-controlled, *RCM* robotic camera mount, *SI* Single-Incision, *SILC* Single-incision laparoscopic cholecystectomy, *SIRC* Single-incision robotic cholecystectomy

A total of 804 urgent cholecystectomies were performed with robotic assistance, 101 of them with an RCM. The remaining 703 patients underwent surgical intervention with an SR. The Da Vinci^®^ system was used in 546 (77.7%) patients; the robot type was not reported for 130 patients. One study explicitly analyzed urgent operations as the main interest [23].

Eleven publications reported cholecystectomies using the single-incision robotic technique (SIRC).

The reports of complications differed greatly, as did the follow-up periods (0–6 months). Kubat et al. showed 12% complications in urgent cholecystectomies, including one (1.5%) biliary tract injury [23]. The highest reported complication rate was 20% (including seromas), and 9 studies stated that no complications occurred. The incidence of Clavien–Dindo grade ≥ 3 complications varied between 1.7 and 4.2%. Biliary tract problems (injury, leakage) were demonstrated in three studies, each with one patient. Hernias represented a particular complication of single-site operations; these were described in three studies, with an incidence of 0.9–6.5%. Conversion to a laparoscopic procedure or to open surgery was reported in 7 studies, and two studies indicated that conversion was necessary due to inflammation [33, 34]. In one study, conversions were an exclusion criterion [35]. Six authors reported a prolonged operation time and one reported a decreased operation time in the robotic group compared to laparoscopic cholecystectomies. One study reported higher costs for robotically operated patients [36].

### Gastrointestinal procedures

Urgent gastrointestinal operations were investigated in 12 studies (Table 3). There were three studies in this group of topics that specifically investigated urgent operations: Anderson et al., Beltzer et al. and, most recently, Robinson et al. [24, 37, 38]. The publication by Ohmura et al. on the use of an RCM evaluated for appendicitis and cholecystitis also reports on the operation of 16 perforations in the upper gastrointestinal tract [14]. Three very similar case reports of Kudsi et al. were combined into a case series [9–11].

**Table 3:** Gastrointestinal procedures

References	Study design						Outcome			Further notes
	Design	Study period	*n* (urgent, robotic)	*n* (non-urgent)	primary study objective	Robot	Differentiated urgent vs. non-urgent	Complications conversions	Further outcomes of interest	
				*n* (other)						
Anderson et al. [24]	R-NRC	2/2015–2/2017	6	–	Urgent subtotal colectomy: robotic vs. Laparoscopic	Da Vinci	Yes	20% (*n* = 1) CD 2	OT longer in robotic	2 dockings
				13				No conversions		
Beltzer et al. [37]	R-NRC	10/2013–11/2018	2	58	Diverticular disease: robotic vs. Laparoscopic	Da Vinci	No	15% cd ≥ 3; 15% < 3	Length of postop ileus in robotic group shorter	Urgency unclear, used: CDD Type 2c
				46				1.7% (*n* = 1) conversions		
Felli et al. [63]	CR	n.d	1	–	Hemicolectomy for hemorrhagic cancer	Da Vinci	Yes	No complications	Double-barreled ileocolostomy; oncologic resection	
				–				No conversion		
Guerra et al. [64]	CR	n.d	1	–	Acute large bowel malignant obstruction	Da Vinci	Yes	No complications	Oncological resection of a splenic flexure tumor	
				–				No conversions		
Kudsi et al. [9–11]	3 CR	2019–2020	3	–	3 colon resections	Da Vinci	Yes	No complications	All with hand sutured anastomosis	3 video vignettes
				–				No conversions		
Ohmura et al. [14]	R-NRC	12/2014–3/2017	16	–	Feasibility	Soloassist RCM	No	“No device-related complications”	LOS shorter liberation of surgical assistant	See also appendicitis and cholecystitis
				933				No conversions		
Pedraza et al. [65]	CR	n.d	1	–	Iatrogenic colonic perforation;	Da Vinci	Yes	No complications	Primary repair	
				–				No conversions		
Robinson et al. [38]	R-NRC	2015–2019	24	–	Perforated gastrojejunal ulcera	Da Vinci	Yes	8.3%, median cd-score 2.2	Complications similar to laparoscopic group	Robotic vs. laparoscopic; higher costs in robotic
				20				No conversions		
Sudan et al. [66]	CR	n.d	1	–	Duodenal stump insufficiency	Da Vinci	Yes	No complications		5 d after BPD-DS, biliary peritonitis
				–				No conversions		
Sun et al. [67]	CR	3/2014	1	–	Gastric perforations	Microhand S	Yes	No complications		
				–				No conversions		
Yi et al. [12]	CS	3/2014	1	–	Repair of gastric perforation	Microhand S	No	No complications		See also appendicitis and cholecystitis
				1				No conversions		
Yi et al. [13]	CS	4/2014–5/2014	1	–	Repair of gastric perforation	Microhand S	No	No complications	Withdrawal of 2 patients	See also appendicitis and cholecystitis
				7				No conversions		

Only statistically significant differences listed

*BPD* biliopancreatic diversion with duodenal switch, *CD* Clavien-Dindo classification of complications [8], *CDD* classification of diverticular disease [39], *CR* Case report, *CS* Case series, *LOS* length of stay, *n.d.* not documented, *OT* Operation time, *RCM* Robotic camera mount, *R-NRC* retrospective, non-randomized, controlled

It remains unclear how many of the 60 robotically operated patients reported by Beltzer et al. were treated in an emergency situation. For this review, we accepted two of the 60 patients with diverticulitis type IIc of classification of diverticular disease (CDD) since they undoubtedly belonged to the group of urgent operations [39]. The study did not distinguish the complications between urgent and elective operations. The overall complication rate was 30%, with percentages of 8.3% for Clavien–Dindo grade 3b complications and 6.7% for anastomotic leakage. One fatality (Clavien–Dindo grade 5), and the need for conversion in another case were described [37]. Anderson et al. listed a complication rate of 20%, though with no further specifications (*n* = 1) [24]. Robinsons’ primary focus was on a typical urgent operation: perforated gastrojejunal ulcers. He reported noninferiority to the laparoscopic approach but dramatically higher costs for robotic operations. None of the case reports/case series reported any complications.

### Hernias/miscellaneous

SRs are commonly used in hernia surgery; however, the urgency of these operations arises from the incarceration of the hernia, which is often associated with intestinal obstruction. We identified eight reports of urgent robotic operations for hernias (Table 4). Four studies retrospectively analyzed urgent hernia operations, two of which were controlled studies [25, 40, 41]. In addition, we identified a database analysis that included urgent robotic hiatal hernia operations [27]. A rare indication for urgent robotic surgery was the operation of posttraumatic splenic bleeding reported by Giulianotti et al. [15]. No reports on the use of an SR in adhesive intestinal obstruction were found.

**Table 4:** Hernias/Miscellaneous

References	Study design						Outcome			Further notes
	Design	Study period	*n* (urgent, robotic)	*n* (non-urgent)	Primary study objective	Robot	Differentiated urgent vs. non-urgent	Complications	Further outcomes of interest	
				*n* (control)				Conversions		
Bou-Ayash et al. [25]	R-NRNC	2/2013–5/2020	19	–	Incarcerated inguinal hernia	Da Vinci	Yes	5.3% CD IVa (*n* = 1; hypercarbia); 10.6% CD < 3		
				–				No conversions		
Cubas et al. [68]	CR	n.d	1	–	Incarcerated morgagni hernia	Da Vinci	Yes	No complication		
				–				No conversion		
Ceccarelli et al. [69]	CS	12/2009–12/2019	3	–	Strangulated hiatal hernias	Da Vinci	Yes	33% (*n* = 1) CD 3		
				2 lap				No conversions		
Giulianotti et al. [15]	R-NRNC	10/2000–11/2002	1	192	Feasibility; here: posttraumatic spleen hematoma	Da Vinci	No	No complications	One CD V in non-urgent patients	
				–				No conversions		
Hosein et al. [27]	database query	2015–2017	131	704	Hiatal hernia repair: robotic vs. Lap. Vs. Open	n.d	No	2% overall complications; 0.1% (*n* = 1) CD V	More urgent and severe ill cases in open; robotic less complication than lap/open	
				1488 open 6774 lap				n.d		
Muhonen et al. [70]	CS	n.d	1	–	Incarcerated paraduodenal hernia	Da Vinci	Yes	No complications		
				2 lap				No conversions		
Muysoms et al. [41]	R-NRC	1/2016–12/2019	8	396	Robotic vs. Lap. Inguinal hernia	Da Vinci	No	3.5% CD II, 0.2% CD IIIb	Shorter hospital stay, higher costs in robotic group	
				272				No conversions		
Kudsi et al. [40]	R-NRC	2013–2019	34	–	Incarcerated hernia robotic vs. Open	Da Vinci	Yes	23.3% < CD 3; 13.3% ≥ CD 3; 3.2% recurrence	OT shorter in open, more CD ≥ 3 in open, more SSI in open	IPOM & TAPP
				43				No conversions		
Smith et al. [71]	CR	n.d	1	–	Incarcerated inguinal hernia	Da Vinci	Yes	No complications		TAPP
				–				No conversion		

Only statistically significant differences listed

*CD* Clavien-Dindo classification of complications, *CS* Case series, *lap* laparoscopic, *IPOM* intraperitoneal only mesh, *n.d.* not documented, *OT* operation time, *R-NRC* non-randomized, controlled, *R-NRNC* retrospective, non-randomized, not controlled, *SSI* surgical site infections, *TAPP* transabdominal pre-peritoneal hernia repair

For urgent robotic hernia operations, the published complication rates were very heterogeneous; the database analysis of Hosein et al. reported one death (0.1%) and an overall complication rate of 2% [27]. Muysoms et al. reported 3.5% minor and 0.2% major complications [41]. Kudsi et al. published a complication rate of 36.6% (23% minor and 13% major complications); however, this rate was significantly lower than that in the open surgery subgroup of Clavien–Dindo grade ≥ 3 complications [26]. A further interesting outcome was the significantly shorter hospital stay but higher costs and the recurrence rate of 3.2% reported by Myosoms et al. [41]. All studies emphasized that the interventions were technically feasible using an SR.

## Discussion

### General considerations and assessment of evidence

To date, there is only limited research on urgent robotic operations in general surgery; therefore, the data available are still very limited. Against this background, we consider a review such as ours, which systematically collects and examines the existing data and presents it descriptively, to be as comprehensive as possible and to be valuable and necessary.

Our review gives clear indications that robotic surgery has not yet arrived in urgent general surgery on a larger scale. A randomized controlled trial has not yet been performed, which is not totally unexpected given that randomized trials dealing with emergency and urgent surgery are generally very rare. However, even the nonrandomized studies offer specific data only to a limited extent: only six studies named urgent operations as a main interest or important variable [23–27, 38]. This makes a systematic assessment of the evidence complicated and a meta-analysis practically impossible. It can be stated that there is only a low level of evidence regarding robotic surgery in the context of urgent operations. Nevertheless, we were able to identify and summarize a notable number of publications covering a wide range of diseases.

Case reports and series are sources with limited evidence and are not suitable for a meta-analysis. In the absence of sources with better evidence, these are nevertheless presented in our study, as these reports fulfill the function of documenting the technical feasibility of certain interventions in the sense of a proof of concept.

With the recently published position paper of the WSES, the topic of urgent robotic operations was highlighted for the first time. De’Angelis et al. also conducted an extensive literature search. The number of papers screened was comparable to our study, but de’Angelis et al. used only ten manuscripts for their analysis: five retrospective cohort studies, and five case reports/case series [4]. We decided against reducing the number of publications used through a stricter assessment since we wanted to provide a maximum amount of information about robotic emergency operations. Nevertheless, our presentation goes beyond a purely narrative review, as, under verifiable conditions, we offer the first complete overview of the published data. However, the conclusions the authors of the WSES statement published are very similar to our findings, though we covered a wider field of operations [4]. The combination of de’Angelis et al. and our work creates, for the first time, a deeper impression of the significance of robotic operations in urgent surgery.

The main indications for robotic emergency interventions were gallbladder, hernias, and gastrointestinal surgery, as well as appendectomies. Some common aspects can be identified. First, there are supposedly higher costs of robotic interventions. Our review shows that robotic operations are significantly more expensive than laparoscopic surgeries [36, 38, 41, 42]. To date, there are no data that demonstrate the amortization of the extra costs by a reduction in the length of stay or complications that stem from the use of an SR. Moreover, the abovementioned studies that showed a cost increase per procedure did not take the considerable acquisition costs of the systems into account. The option of refunding by additional charges for the use of an SR is not possible in all health systems. Second, an often cited counterargument against robotic emergency interventions is the increased time requirements due to longer preparation or operation times. Here, the data were ambiguous and ranged from lengthening to shortening of time intervals with a tendency toward increased operative time. Minimally invasive surgery is primarily intended for stable patients, which of course also applies to robotic operations. Thus, even the moderate increases in operating time described above do not seem to be a contraindication for robotic operations.

### Robotic camera mounts (RCM)

RCMs can be classified as robotic surgery only to a limited extent. However, they offer some of the alleged advantages, in particular a stable image and ergonomic advantages. However, there are also might be disadvantages, e.g., complications due to technical malfunctions.

According to this review, this type of surgery seems to be not very widespread, especially in urgent surgery, as only two publications have described the use of these systems [14, 21].

No clear statements about the advantages and disadvantages of RCMs can be drawn from the data presented here, but it can be stated that this type of surgery is undoubtedly more similar to laparoscopic than to robotic surgery. However, it seems important that no RCM-associated complications are reported.

### Appendectomies

Appendectomy is a very frequent operation, although there have been very few studies that deal with robotic operations for acute appendicitis. This is of particular interest since appendicitis was one of the diseases that significantly led to the development of modern laparoscopy, initially starting with the confirmation of the diagnosis and the first laparoscopic appendectomy by Semm in 1983 [43]. Today, laparoscopic appendectomy is the therapy of choice for appendicitis [2].

There may be various reasons for the lack of studies on SR for appendicitis: The presumed high costs of robotic operations have been discussed since the start of robotic surgery [30]. Most likely, a “minor” procedure such as an appendectomy with correspondingly low remuneration is estimated as economically unreasonable and will therefore not become part of clinical routine for robotic surgery. It is also conceivable that the advantages of robotic operations for an often rather simple operation without complex preparation are not regarded as sufficient to implement this technique. An urgent appendectomy with the Da Vinci^®^ system was reported in only two cases, while this robot system has been generally used most often. The feasibility of appendectomies with the Da Vinci^®^ system was shown by several studies reporting on appendectomies in the context of other operations [44, 45].

An option to make a robotic appendectomy economically worth considering is to overthink the closure of the appendix stump: the use of the stapler or clip applicator for the Da Vinci^®^ system causes relevant costs. This could be circumvented by closing the stump with a Roeder loop or suture. However, a current meta-analysis showed that stump closure with staples is superior in terms of postoperative complications [46].

In the overall view, appendectomy via SR is technically possible, but the additional expense compared to laparoscopic appendectomy most likely cannot be justified.

### Cholecystectomies

Regarding this indication, the number of published studies is better, although far from satisfactory. However, against the background of more available studies, a case report analysis was less necessary. In addition to the fact that one of these case reports was among the first publications on robotic surgery in general surgery (Hanisch et al.), these reports did not provide any crucial information [30]. The statements regarding the advantages and disadvantages of the robotic approach in urgent cholecystectomies were very heterogeneous without a clear trend.

Remarkably, many studies compared single-incision robotic cholecystectomy (SIRC) with nonrobotic surgical procedures. Therefore, we assume a reporting bias: multi-incision robotic cholecystectomy (MIRC) was primarily examined in studies that were published before 2010, and acute cholecystitis was often an exclusion criterion [19]. However, the scientific perception of robotic cholecystectomy may have changed in two aspects: MIRC appears to be such a standard procedure that it is less examined in studies. At the same time, and based on their increased robotic experience, more researchers have opted to include acute cholecystectomy in their studies. In our estimation, these interventions are currently often performed as SIRC since this technique is assumed to be more innovative. Furthermore, there is another inaccuracy in the analysis: a number of studies reported robotic surgery for acute cholecystitis, with acute inflammation diagnosed intraoperatively. Therefore, this might not be labeled “urgent surgery” correctly in the narrower sense. The subject of this analysis is not the question of single- vs. multi-incision operations. The currently most up-to-date Practice Guideline on Safe Cholecystectomy votes for MIRC, in particular due to an increased rate of biliary tract injuries in the single-incision group [47]. Regarding the literature analyzed in our study, there was one biliary tract injury during an operation for acute cholecystitis using the SIRC technique. The data on complications and conversions were heterogeneous, as were the statements on the possible advantages and disadvantages of robotic operations. Biliary tract problems were reported in three patients, which resulted in a biliary complication rate of 0.4% among all patients operated on with an SR, which was within the expected range. Except for one study, these complications were not differentiated between urgent and elective operations. Notably, Kulaylat et al. reported an increase in hospital costs of 38% for the robotic procedure compared to laparoscopic cholecystectomy [36]. In summary, at the moment, neither the advantages nor the risks of robotic operations in urgent, acute cholecystectomies can be adequately assessed. However, no clear contraindications for the use of robots in this situation were found in this review.

### Gastrointestinal procedures

The advantage of robotic surgery in acute conditions of the gastrointestinal tract, especially perforations, seems obvious: since a robot provides a significant advantage for suturing and tying knots, perforations and ruptures can be closed excellently. Furthermore, robotic operations for benign and malignant diseases of the colon or the upper gastrointestinal tract are clinical routine, with an increasing proportion being performed in the elective setting [48]. Our review shows that for urgent operations, there are hardly any data available. However, our own experience and the case reports/series listed demonstrate that these interventions were definitely possible in an urgent setting.

Beltzer et al. summarized that there was no advantage in using the robot for surgical procedures in diverticular disease [37]. Anderson et al. also did not state any explicit advantages and reported a longer operation time [24]. There are currently no data that propagate the use of SR in this indication group for a better outcome in a population operated urgently.

Robinson et al. presented a study primarily focused on urgent robotic surgery for perforated gastrojejunal perforations [38]. The authors were able to show that robotic surgery was not inferior to laparoscopic surgery. In addition, they reported two interesting aspects that otherwise hardly received attention: (1) the immediate preparation time in the OR was even shorter than that with laparoscopic operations; and (2) 54% of the operations were performed on weekends or during the evening, night or early morning. This invalidates some of the arguments that have been put forward against robotic emergency operations.

As mentioned above, in regard to surgery of the gastrointestinal tract, the cost disadvantages of SRs matter again: stapler and sealing devices in addition to draping, scissors, forceps, etc., are significant cost factors. Schiergens et al. showed that the use of an SR for an elective sigma resection increases the cost of surgical supplies by more than a factor of 4 compared to open surgery and by more than a factor of 2 compared to laparoscopic surgery. These numbers were very comparable to the cost increase reported by Robison et al. for urgent gastrojejunal ulcers [38, 49].

### Hernias/miscellaneous

SRs are used regularly in minimally invasive hernia surgery [3]. This may contribute to the fact that there are quite a few studies on urgent robotic hernia surgery. Even if the evidence cannot be described as satisfactory, the overall picture is similar to those of the other indication groups: robotic interventions are feasible in incarcerated hernias and in urgent situations. The complication and conversion rates were low and comparable to those of nonrobotic, minimally invasive procedures. To date, however, no clear advantages of the robotic technique have been demonstrated.

The situation is different in hiatal hernias: in most cases, hiatal hernias are technically more demanding than inguinal or ventral hernias. The sutures required to reconstruct the hiatus and to create the fundoplication as well as the preparation make this operation ideal for the use of an SR. Current studies showed fewer complications and a shorter hospital stay for robotic hiatus hernia surgery in the elective setting [50]. Even if no data are available for urgent hiatoplasty, it is quite likely that such effects can be observed here.

The report of a splenectomy for bleeding (along with several reports of elective splenectomies) indicated that this operation is also possible with an SR. If the patient’s circulatory system remains stable and a minimally invasive procedure is conceivable, the procedure can also be carried out with an SR if the appropriate expertise is available.

## Conclusion

A particular value of our work is that it provides a well-founded summary of the existing data for surgeons and researchers who are interested in urgent abdominal robotic surgery. Based on this, the specific studies needed can be initiated in the future. Furthermore, our review may help to establish framework conditions for a register for urgent robotic interventions.

With this review, we provide the most complete overview of the current literature on robotic surgery for urgent general surgical operations. Our analysis of the literature gives the impression that, in particular, robotic cholecystectomies in acute cholecystitis and operations on impacted hernias are feasible and rational and can be carried out without increased risk. Initial studies on urgent robotic operations in the gastrointestinal tract have thus far shown ambivalent results but have been proven technically feasible. High costs remain a significant burden for these operations.

## Search algorithm

#1 (“robot*”[MeSH Terms] AND “robot*”[Title/Abstract]) OR “robotic surgery” OR “robot-assisted” OR “robot assisted”

#2 #1 AND adhesiolysis

#3 #1 AND (appendicitis OR append*)

#4 #1 AND bowel obstruction

#5 #1 AND (cholecystitis OR cholecystectomy OR cholecyst*)

#6 #1 AND diverticular disease

#7 #1 AND diverticulitis

#8 #1 AND “emergency”

#9 #1 AND “emergencies”

#10 #1 AND hernia

#11 #1 AND (incarcerated OR incarcerat*)

#12 #1 AND “ischaemia”

#13 #1 AND perforation

#14 #1 AND peritonitis

#15 #1 AND ulc*

#16 #1 AND urgency

#17 #1 AND urgent

#2 OR #3 OR #4 OR #5 OR #6 OR #7 OR #8 OR #9 OR #10 OR #11 OR #12 OR #13 OR #14 OR #15 OR #16 OR #17
